# Effects of Positive Airway Pressure Therapy on Cognitive Function in Patients with Obstructive Sleep Apnea: A Prospective Observational Study Protocol

**DOI:** 10.3390/life16040692

**Published:** 2026-04-21

**Authors:** Branislav Kollár, Stela Biathová, Katarína Klobučníková, Peter Turčáni, Žofia Rádiková, Ingrid Žitňanová, Ľubica Argalášová, Pavel Šiarnik

**Affiliations:** 1 1st Department of Neurology, Faculty of Medicine, Comenius University and University Hospital Bratislava, 813 69 Bratislava, Slovakia; branislav.kollar@fmed.uniba.sk (B.K.); katarina.klobucnikova@fmed.uniba.sk (K.K.); peter.turcani@fmed.uniba.sk (P.T.); pavel.siarnik@fmed.uniba.sk (P.Š.); 2 Institute of Clinical and Translational Research, Biomedical Research Center, Slovak Academy of Sciences, 845 05 Bratislava, Slovakia; zofia.radikova@savba.sk; 3 Faculty of Health Sciences, University of Ss. Cyril and Methodius in Trnava, 917 01 Trnava, Slovakia; 4 Institute of Medical Chemistry, Biochemistry, and Clinical Biochemistry, Faculty of Medicine, Comenius University, 811 08 Bratislava, Slovakia; ingrid.zitnanova@fmed.uniba.sk; 5 Institute of Hygiene, Faculty of Medicine, Comenius University, 813 72 Bratislava, Slovakia; lubica.argalasova@fmed.uniba.sk

**Keywords:** obstructive sleep apnea, cognitive impairment, observational cohort study

## Abstract

Obstructive sleep apnea (OSA) is the most common sleep-related breathing disorder and is increasingly recognized as a contributor to cognitive decline and a potential risk factor for neurodegeneration. Previous studies have also identified various associated comorbidities such as vascular dysfunction, metabolic alterations, and neuroinflammatory changes. Positive airway pressure (PAP) therapy has been associated with cognitive improvement in some studies, but its long-term effects on cognitive function remain uncertain. This study employs a prospective, observational, longitudinal cohort design to examine longitudinal associations between disease severity, PAP therapy and cognition. Additionally, we aim to examine the relationships between cognitive dysfunction, brain structure and associated OSA-related risk factors. A total of 100 eligible participants with mild to severe OSA will be recruited. All participants will undergo comprehensive assessments at baseline and after 12 months, including neurological, pulmonary, and ear, nose and throat clinical examinations, polysomnography, neuropsychological testing, brain magnetic resonance imaging with volumetry, anthropometric measurements, blood and saliva sampling for the assessment of the selected laboratory parameters, gut microbiome analysis, and evaluation of endothelial function and baroreflex sensitivity. This study may improve understanding of how PAP therapy and OSA-related pathophysiological processes influence cognitive outcomes.

## 1. Introduction

Obstructive sleep apnea (OSA) is a multifaceted sleep disorder characterized by repeated episodes of upper airway obstruction during sleep, leading to significant reductions in airflow and oxygenation. This condition manifests as complete cessation of breathing (apneas) lasting 10 s or more, or partial obstructions (hypopneas) that result in reduced airflow [1,2]. Beyond its primary respiratory manifestations, OSA is associated with major comorbidities such as cardiovascular, metabolic, and neurocognitive disorders, affecting overall health and quality of life [3,4,5,6]. Recent research has also begun to focus on the interplay between OSA and biomarker-related susceptibility to neurodegeneration, particularly highlighting associations with the apolipoprotein E (ApoE) ε4 allele and plasma neurofilament light chain (pNfL) levels. The presence of the ApoE ε4 allele has been linked to increased vulnerability to OSA-related cognitive decline [7,8,9], while elevated levels of pNfL may indicate axonal injury and neurodegeneration in affected individuals [10,11,12].

With the global rise in dementia and aging populations, identifying modifiable contributors to cognitive decline is critical [13]. OSA affects nearly one billion people globally, yet remains underdiagnosed—particularly in older adults, where its cognitive effects may overlap with or accelerate early neurodegenerative changes [14,15,16]. As a treatable condition, OSA may play a key role in preventing or delaying cognitive impairment in vulnerable populations.

While a number of studies have examined the effects of positive airway pressure (PAP) therapy on cognitive function and brain structure [17,18,19], to our knowledge no study to date has explored the full range of comorbidities and biological mechanisms relevant to cognitive outcomes in OSA. Moreover, most existing studies have used brief screening tools, limiting sensitivity to subtle or domain-specific impairments in OSA. This limited scope has constrained our understanding of the complex, multifactorial effects of PAP therapy on the brain. In this study, we aim to provide a detailed description of the cognitive profile, investigate the association between disease severity, PAP therapy and cognition, and explore the relationships between OSA and its contributing factors.

## 2. Materials and Methods

The design of this study protocol was developed in accordance with the STROBE guidelines [20] for observational studies. Selected elements of the SPIRIT 2013 guidelines [21] were also considered where applicable. This study was registered on ClinicalTrials.gov (Identifier: NCT07364318).

### 2.1. Study Aims and Hypotheses

The primary aim of this study is to perform a detailed cognitive assessment in patients with OSA. We aim to analyze the baseline prevalence, severity, and pattern of cognitive deficits in this population and to evaluate changes in cognitive function after 12 months of PAP therapy to gain a clearer understanding of the initial cognitive status from which potential therapeutic or disease-related changes may occur.

The secondary aims of this study are exploratory. We plan to investigate associations between cognitive outcomes and a broad spectrum of OSA-related measures, including polysomnography (PSG)-derived sleep parameters, brain volumetric measures, anthropometric and metabolic indicators, biomarkers (e.g., pNfL, oxidative stress markers, lipid subfractions, ApoE genotype), gut microbiome composition, vascular function, and autonomic nervous system regulation. We also aim to explore how PAP adherence relates to longitudinal changes in these cognitive and physiological outcomes.

### 2.2. Study Design and Setting

This study employs a prospective, observational, longitudinal cohort design, conducted at the Sleep Laboratory of the 1st Department of Neurology, Faculty of Medicine, Comenius University, University Hospital Bratislava, Slovakia. The study will enroll at least 100 eligible participants with newly diagnosed mild to severe OSA, who have no history of chronic diseases (except for overweight and obesity) and are not receiving any chronic medication. The Sleep Laboratory has active research programs on sleep disorders, an established infrastructure for clinical studies, and a high volume of patient referrals. Participants meeting all inclusion criteria will receive detailed information about the purpose and procedures of the study before enrollment.

Diagnosis of OSA must first be confirmed by overnight PSG. Participants will then enter the cohort after meeting the inclusion/exclusion criteria described below. Trained research personnel will obtain informed consent from the patient after providing all the necessary information. Cognitive function, OSA-related measures (PSG variables), sleep parameters, brain magnetic resonance imaging (MRI) and volumetric measures, anthropometric parameters, blood and saliva biomarkers, gut microbiota composition, endothelial function, and baroreflex sensitivity will be assessed at baseline and after 12 months, together with neurological, pulmonary, and ear, nose, and throat (ENT) examinations. Those patients who are routinely clinically indicated for PAP therapy will receive treatment, and their adherence will be monitored throughout the study. PAP therapy is prescribed exclusively based on routine clinical indications and follows standard-of-care pathways. The study protocol does not mandate initiation, modification, or discontinuation of PAP therapy and does not include any study-driven therapeutic decision-making. Participants are not randomized to treatment groups, and no experimental group allocation is performed. Blinding of participants and investigators to PAP therapy status is not feasible due to the nature of the study design. Participants are invited to contact the principal investigator at any time to inquire about results. The general study timeline is described in Table 1.

### 2.3. Eligibility Criteria

#### 2.3.1. Inclusion Criteria

1. Signed informed consent;
2. Age between 18 and 65 years;
3. Newly diagnosed OSA according to the American Academy of Sleep Medicine Guidelines (overnight PSG with an apnea-hypopnea index (AHI) ≥ 5 events per hour, hypopneas defined by ≥10 s of airflow reduction accompanied by ≥3% desaturation or an arousal) [21];
4. Ability to complete all relevant tests and follow-up visits.

#### 2.3.2. Exclusion Criteria

1. History of any chronic disease other than overweight and obesity;
2. Severe psychiatric condition that could affect cognitive functions;
3. Chronic use of medication or nicotine that may affect the study results;
4. Prior neuropsychological assessment performed less than 6 months before study enrollment;
5. Prior therapy for OSA (e.g., PAP, upper airway surgery, oral appliance);
6. Motor or sensory deficits that would significantly complicate test administration;
7. Patients with insufficient Slovak language proficiency for valid participation in the study;
8. Considered by the study team as not suitable for enrollment based on clinical judgment.

#### 2.3.3. Withdrawal Criteria

If a patient with a stable medical condition at enrollment experiences a deterioration of the existing condition or develops a new condition, the participant will be assessed by the study physician, and the treating physician(s) will be consulted. If either the study physician or the treating physician considers continued participation inadvisable, the participant will be immediately withdrawn from the study and referred back to their treating physician for appropriate management. Given the observational nature of the study and the use of standard-of-care procedures, no study-specific risks or harms are anticipated.

### 2.4. Baseline Measures, Exposures and Confounders

#### 2.4.1. Polysomnography

PSG (using the Alice 6 Philips-Respironics device, Murrysville, PA, USA) represents a laboratory examination simultaneously recording vital functions such as sleep/wakefulness, breathing, blood oxygen saturation, heart activity, limb movements, etc. It will be performed on all patients who are hospitalized in the Sleep Laboratory of the 1st Department of Neurology with suspicion of OSA to confirm or exclude this diagnosis, and in patients with confirmed OSA, to characterize the severity and specific features of the disorder. The sleep examination will include a questionnaire-based assessment of daytime sleepiness (Epworth Sleepiness Scale) and sleep quality (Pittsburgh Sleep Quality Index).

#### 2.4.2. Neuropsychological Assessment

The cognitive evaluation will focus on core cognitive domains and will include tests from the NEUROPSY battery adapted for the Slovak population: (1) Rey auditory—verbal learning test, (2) Controlled oral word association test, (3) Category fluency test, (4) Trail-making test, (5) Stroop color and word test, (6) Digit span and from short Czech neuropsychological battery, (7) Figure copy task. Based on the results, the presence of cognitive dysfunction will be determined. Further questionnaire-based assessments will be conducted, including the Generalized anxiety disorder 7-item scale (GAD-7), Patient health questionnaire-9 (PHQ-9), and screening of cognitive functions through the Montreal cognitive assessment (MoCA). The MoCA will be included as a brief screening tool to allow external comparability with other studies but will not substitute for the comprehensive battery.

*A detailed description of each cognitive test and questionnaire is provided in Appendix A (Table A1). All participants complete the tests in a standardized order as listed.*

#### 2.4.3. Brain Magnetic Resonance Imaging and Volumetry

MRI of the brain will be performed using a PHILIPS Ingenia 3T Omega HP scanner (Philips Healthcare, Best, The Netherlands). Standard sequences will include T1- and T2-weighted imaging, FLAIR sequences in axial, coronal, and sagittal planes, diffusion-weighted imaging (DWI), apparent diffusion coefficient (ADC), and susceptibility-weighted imaging (SWI). Brain volumetric analysis will be performed using the icobrain software (version 5.16.6; icometrix NV, Leuven, Belgium), enabling quantitative assessment of total and regional brain volumes, including total brain volume, gray and white matter volumes, hippocampal volume, cortical thickness, and other selected regional structures relevant to cognitive function (units: mL or mm^3^; volume changes reflect structural brain alterations).

#### 2.4.4. Anthropometric Measurements

Anthropometric assessment will focus on the evaluation of overweight and obesity. Body mass index (BMI) will be calculated as weight in kilograms divided by height in meters squared. Body composition will be assessed using bioelectrical impedance analysis (BIA) with Omron and InBody devices. A comprehensive analysis of body composition, including fat mass, fat-free mass, and body fat percentage, will be performed using the InBody 720 device (Biospace, Seoul, Republic of Korea), which utilizes direct segmental multi-frequency BIA. In addition, body fat estimation will be obtained using skinfold thickness measurements with calipers. Daily physical activity will be assessed using the Slovak version of the Lagerros questionnaire.

#### 2.4.5. Blood and Saliva Sampling

Venous blood and saliva samples will be collected for laboratory analyses. In blood, we will assess lipid and glucose metabolism, inflammatory, neuronal, and metabolic markers, metalloproteinase activity, and hypoxia-related gene expression in peripheral blood mononuclear cells (PBMCs). Oxidative stress and selected metabolic and lipid markers will be analyzed in both blood and saliva.

The examination of the lipoprotein profile will involve determining the levels of total cholesterol, low density lipoprotein (LDL) cholesterol, high density lipoprotein (HDL) cholesterol, and triglycerides in a certified laboratory using an autoanalyzer (Siemens Healthcare Diagnostics Inc., Tarrytown, NY, USA) following standard procedures with enzymatic kits (Roche Diagnostics, Lewes, UK). Detailed lipid metabolism with a focus on the determination of HDL and LDL subfractions of lipoproteins in plasma will be analyzed using electrophoresis on a polyacrylamide gel system called Lipoprint (Quantimetrix Corporation, Redondo Beach, CA, USA).

The role of oxidative stress will be analyzed by monitoring plasma and saliva antioxidant capacity (spectrophotometrically) and levels of markers of oxidative damage to lipids (lipoperoxides) (spectrophotometrically). All markers will be determined in both plasma and unstimulated whole saliva.

The role of endothelial function, metabolic regulation, and systemic inflammation will be assessed by measuring plasma levels of asymmetric dimethylarginine (ADMA), adiponectin, and lipopolysaccharide-binding protein (LBP), respectively. ADMA serves as an endogenous inhibitor of nitric oxide synthase and reflects endothelial dysfunction. Adiponectin is a key adipokine involved in glucose and lipid metabolism with anti-inflammatory properties, and LBP is a marker of bacterial endotoxin exposure and immune system activation. All markers will be quantified using validated immunoassays according to the manufacturers’ protocols.

PBMCs will be isolated from 15–20 mL of blood using Ficoll Paque Plus gradient centrifugation and stored at −80 °C in CryoStor medium (BioLife Solutions, Inc., Bothell, WA, USA) until nucleic acid extraction. Plasma will be collected from each sample for zymographic determination of matrix metalloproteinase activity. PBMCs will be used to monitor mRNA expression of selected genes using reverse transcription quantitative polymerase chain reaction (RT-qPCR) with TaqMan probes (Thermo Fisher Scientific Inc., Waltham, MA, USA). Relative gene expression will be calculated from CT values using Bio-Rad CFX Manager software (version 3.1; Bio-Rad Laboratories, Hercules, CA, USA).

ApoE genotyping will be performed to investigate genetic predisposition to neurodegenerative and vascular changes.

#### 2.4.6. Gut Microbiome from Stool

Stool samples will be collected in sterile tubes for cultivation and in DNA/RNA Shield tubes (Zymo Research, Irvine, CA, USA) for nucleic acid preservation. Gut microbiome characterization will be performed using up-to-date methods, including 16S rRNA V3–V4 region metagenomic sequencing on the Illumina MiSeq system with commercially available library and sequencing kits (BiovendorMDx (BioVendor—Laboratorní medicína a.s., Brno, Czech Republic); MiSeq^®^ Reagent Kit v3 (Illumina, Inc., San Diego, CA, USA)). Sequencing data will be processed using established bioinformatics pipelines, including quality filtering, read assembly, and taxonomic assignment. Downstream analyses will include assessment of microbial composition as well as alpha and beta diversity metrics. In vitro cultivation of gut microbiota will be performed to complement sequencing-based analysis, enabling quantitative and functional assessment of viable bacterial populations. The combination of cultivation and flow cytometry will allow high-resolution identification and quantification of bacterial species within complex communities. Additionally, culture-based analyses will provide insight into microbial functional outputs, including the production of extracellular vesicles and extracellular RNA, such as miRNA.

#### 2.4.7. Vascular Functions

Subclinical atherogenesis will be examined using peripheral arterial tonometry (PAT) with the EndoPAT device (Itamar Medical Ltd., Caesarea, Israel). The examination is performed non-invasively with sensors placed on the second finger of both hands. One limb is used to determine resting arterial tonometry, while occlusion of blood flow in the brachial artery with a cuff pressure monitor is induced on the other limb, followed by the observation of reactive hyperemia. The reactive hyperemia index (RHI), a parameter of endothelial function, and the augmentation index (AI), a parameter of arterial stiffness, will be determined.

#### 2.4.8. Baroreflex Sensitivity

The function of the autonomic nervous system (baroreflex sensitivity) will be measured using a non-invasive system, the Finometer^®^ PRO (Finapres Medical Systems B.V., Enschede, The Netherlands), which continuously records blood pressure and pulse frequency values on the fingers of the hand. Autonomic reactivity will be evaluated from spontaneous blood pressure fluctuations.

### 2.5. Outcome Measures

#### 2.5.1. Primary Outcome Measures

##### Cognitive Function

The primary outcome of the study is cognitive function assessed by a comprehensive neuropsychological battery. Primary cognitive outcomes will be derived from test performance across the main cognitive domains including memory, executive functions, attention, language, and visuoconstructional abilities. Cognitive performance will be evaluated both cross-sectionally at baseline, to characterize the prevalence, severity, and pattern of cognitive deficits in patients with OSA, and longitudinally as change in cognitive performance from baseline to 12 months. PAP therapy adherence will be treated as an exposure variable in analyses of longitudinal cognitive change.

To improve comparability across tests and reduce measurement variability, standardized z-scores will be calculated for each neuropsychological measure and subsequently aggregated into domain-specific composite scores. In addition to statistical significance, cognitive outcomes will also be evaluated for clinical relevance. Clinically meaningful improvement will be defined as a change greater than 1 standard deviation (SD) from baseline in domain-specific scores or a shift from impaired to normative performance range (e.g., from ≤5th percentile to >15th percentile) [22]. This ensures that changes in test performance reflect not only statistical but also functional significance.

#### 2.5.2. Secondary Outcome Measures

##### Changes in Baseline Measures

After 12 months of PAP treatment, patients will undergo the same assessments as during their initial hospitalization (besides ApoE genotyping), following the original protocol. This will include overnight PSG, neuropsychological testing, brain imaging, brain volumetry, anthropometric measurements, blood and saliva samples, gut microbiome, vascular functions and baroreflex sensitivity assessments. All collected data will be compared with baseline values, allowing us to explore the associations between PAP therapy and changes in cognitive and other selected parameters. All secondary analyses are considered exploratory and hypothesis-generating.

#### 2.5.3. Exposure Variables of Interest

##### PAP Therapy and Adherence to Treatment

The gold standard treatment of moderate/severe OSA is positive airway pressure (PAP) therapy, which works by holding the airway open using a pneumatic splint. There is evidence to suggest that compliant PAP therapy use can have beneficial effects on some of the consequences of this disorder. Adherence to the treatment will be measured through an adherence tracking system using smart memory cards, which will report all needed parameters. Adherence will be primarily analyzed as a continuous variable (e.g., average hours of use per night) to capture potential dose–response relationships with cognitive outcomes. In addition, secondary analyses will examine adherence as a categorical variable using standard criteria to classify participants as adherent or non-adherent (e.g., ≥4 h of use per night [23]), allowing for clinically interpretable group comparisons. To promote participant retention and complete follow-up, study assessments are scheduled in alignment with routine clinical visits where feasible.

### 2.6. Sample Size Calculation and Power Calculations

Data collection will take place from November 2024 to September 2027 at the latest. If the targeted sample size N = 100 is reached before the end date, data collection will continue until the end of the funding.

Based on preliminary effect sizes reported in prior studies investigating PAP effects on cognition in OSA [18,19], a medium effect size (Cohen’s d = 0.5) is anticipated for within-subject changes in cognitive performance. Using G*Power 3.1.9.7, a total sample size of 34 participants is required to achieve 80% power at α = 0.05 using a two-tailed paired-samples *t*-test. This paired-samples framework provides an approximation of the within-subject effect that will be tested using linear mixed-effects models. After adjusting for an expected 15% attrition rate, this increases to 40 participants.

To support secondary analyses involving multivariable modeling, the target sample size was determined using power analyses for both the primary within-subject comparison and the planned multivariable models. The calculations assumed a medium effect size (Cohen’s f^2^ = 0.15) and up to four predictors. Accounting for an expected 15% attrition rate over the 12-month follow-up, approximately 100 participants will be recruited to ensure sufficient statistical power for all planned analyses.

The final target sample size of 100 participants was determined based on the multimodal nature of the dataset, requiring sufficient sample size for stable model estimation while limiting the number of predictors to reduce the risk of overfitting. Although the minimum required sample for detecting the expected within-subject effect is smaller, the planned sample improves the robustness of the primary outcome analysis, increases resilience to missing data, and allows for reliable adjustment for key covariates.

### 2.7. Data Analysis Plan

Descriptive statistics will be used to summarize demographic and clinical characteristics. The normality of continuous variables will be assessed using visual inspection (Q–Q plots, histograms) and statistical tests (e.g., Shapiro–Wilk). Continuous variables will be presented as mean ± SD for normally distributed data or median with interquartile range for non-normally distributed data. Categorical variables will be expressed as frequencies and percentages.

Primary analyses will evaluate within-subject changes in cognitive function over the 12-month period (i.e., change scores ΔT12–T0). These changes will be analyzed using linear mixed-effects models with a random intercept, which allow for repeated measurements and account for inter-individual variability. Fixed effects will include time, PAP adherence, and covariates such as age, sex, and education. An interaction term between time and adherence will be used to assess whether longitudinal changes differ according to adherence level.

Secondary analyses will explore associations between changes in cognitive outcomes and other measured variables, including sleep parameters, volumetric measures, anthropometric and metabolic indicators, biomarkers, and other selected laboratory measures (e.g., pNfL, ApoE genotype, gut microbiome composition), as well as indices of vascular health and autonomic nervous system function. These associations will be examined using Pearson or Spearman correlations between change scores (ΔT12–T0) and multiple linear regression models with a limited number of predictors (maximum of 4 per model) to minimize the risk of overfitting.

This study is powered to detect medium-sized effects in primary outcomes. Secondary analyses are considered exploratory and hypothesis-generating. The statistical plan will focus on core outcomes, limit the number of predictors per model, and adjust for multiple testing where applicable to minimize the risk of Type I error. Where appropriate, adjustments for multiple comparisons will be applied using procedures such as the false discovery rate (FDR) or other suitable methods. Post hoc comparisons, including subgroup analyses or alternative outcome definitions (e.g., cognitive status, biomarker levels), will be clearly identified as exploratory and interpreted with appropriate caution. Model assumptions will be assessed using standard diagnostic procedures, including evaluation of residual normality, homoscedasticity, and overall model fit. Analyses will be conducted using SPSS (version 27.0.1) or R (version 4.3.1).

### 2.8. Data Collection and Management

Upon enrollment, each participant will be assigned a unique study ID to ensure pseudonymization of personal data. The data in this trial will be collected using traditional print-based case report forms. All data entries will be manually transcribed into the electronic system by trained study personnel. Double data entry will be employed to minimize transcription errors and ensure accuracy. The data storage is achieved using an electronic data capture system (REDCAP) which is HIPAA-compliant and allows storage of data in password-protected files in the cloud. Data acquired in paper form are kept in locked files at the respective study site. Only project staff will have access to the trial dataset. Participants who discontinue PAP therapy or deviate from the protocol will remain in the analytical sample, and all data collected up to the time of discontinuation, including cognitive, clinical, and physiological measures, will be included in the analyses.

## 3. Results

At the time of manuscript submission, participant enrolment is ongoing. Baseline and 12-month follow-up assessments are being conducted according to the protocol described above. The planned analyses will evaluate longitudinal changes in cognitive outcomes and their associations with PAP adherence and other physiological parameters.

## 4. Discussion

As the population ages, more adults experience stroke, cognitive impairment, and dementia. Because no effective treatments currently exist to prevent or slow dementia, identifying modifiable risk factors such as OSA is increasingly important [24].

OSA has been linked to substantial cognitive, metabolic, and cardiovascular consequences [3,4,5,6], yet the extent to which these impairments are reversible with long-term therapy remains unclear. By combining a neuropsychological assessment, physiological measures, neuroimaging, and biomarkers, this study provides a systems-level approach to understanding the pathophysiological mechanisms of untreated OSA and the therapeutic potential of PAP therapy.

Previous meta-analytic findings suggest that cognitive impairment in OSA is domain-specific, with the most consistent deficits observed in executive functions, attention, and information processing speed, while other domains such as memory may be less consistently affected. However, the relationship between OSA severity and cognitive outcomes appears heterogeneous and not strictly linear. Methodologically, many existing studies have relied on brief cognitive screening tools and relatively short durations of PAP therapy, which may limit the detection of subtle or domain-specific changes [4]. Complex neuropsychological assessment both before and after intervention may provide stronger empirical support for the connection between OSA and cognitive impairment or reveal new associations. Through PAP treatment, we aim to investigate whether its use is linked to functional brain changes. To our knowledge, no previous study has investigated these relationships with such an extensive multi-modal assessment and controlled set of underlying pathophysiological mechanisms.

This protocol describes a prospective, observational, longitudinal cohort study. The observational design improves ecological validity by reflecting real-world patient characteristics; however, it also introduces methodological limitations that must be considered. Due to the non-randomized design and absence of a placebo or untreated control group, confounding variables cannot be fully eliminated, and causal inferences will be limited. Given the observational design, confounding by indication cannot be excluded. Although PAP therapy adherence will be objectively measured, substantial inter-individual variability in use may limit the interpretability of treatment effects. To partly address this limitation, we will perform stratified and regression-based analyses according to PAP therapy adherence levels, enabling us to explore dose–response relationships while adjusting for relevant covariates. The intentionally restrictive inclusion and exclusion criteria may raise concerns regarding both selection bias and feasibility. While these criteria were designed to reduce the influence of major confounding factors and enhance internal validity, they may result in a more selected study population and limit generalizability. However, based on preliminary analyses of patient flow at the study site, recruitment of the target sample size is considered achievable despite these constraints. The comprehensive nature of the study protocol may increase participant burden; however, the majority of assessments are part of routine clinical practice in the evaluation of patients with OSA.

In summary, this study aims to take a comprehensive approach to understanding how PAP therapy may affect cognition in OSA through an extensive assessment of various physiological and pathophysiological mechanisms. Our findings may inform future intervention strategies and improve our understanding of reversible contributors to cognitive decline.

## 5. Conclusions

This protocol outlines a multimodal observational study designed to examine the relationships between OSA, cognitive dysfunction, and associated physiological and biological mechanisms. The findings may contribute to a better understanding of how PAP therapy and OSA-related pathophysiological processes are associated with cognitive outcomes.

## Figures and Tables

**Table 1:** Timeline of the study.

Measures	Study Phase			
	T0. Recruiting and Enrollment	T1. Baseline Assessment	T2. Treatment Period	T3. Follow-Up After 12 Months
Informed consent	X			
Demographic data		X		X
Clinical history		X		X
Neurological, pulmonary, and ENT clinical examinations		X		X
Polysomnography		X		X
Neuropsychological assessment		X		X
Brain MRI with volumetry		X		X
Anthropometric measures		X		X
Blood and saliva sampling		X		X
Gut microbiome from stool		X		X
Vascular functions		X		X
Baroreflex sensitivity		X		X
Adherence to PAP therapy (if applicable)			X	X

Abbreviations: ENT—ear, nose, throat; MRI—magnetic resonance imaging; PAP—positive airway pressure.

## Data Availability

The ongoing study includes sensitive clinical data. The data are available from the corresponding author on reasonable request, subject to ethical and institutional restrictions.

## Abbreviations

The following abbreviations are used in this manuscript:

AHI	Apnea-hypopnea index
AI	Augmentation index
ApoE	Apolipoprotein E
BIA	Bioelectrical impedance analysis
BMI	Body mass index
DWI	Diffusion weighted imaging
ENT	Ear, nose and throat
GAD-7	Generalized anxiety disorder 7 item scale
HDL	High density lipoprotein
LDL	Low density lipoprotein
LBP	Lipopolysaccharide binding protein
MoCA	Montreal cognitive assessment
MRI	Magnetic resonance imaging
OSA	Obstructive sleep apnea
PAP	Positive airway pressure
PAT	Peripheral arterial tonometry
PBMC	Peripheral blood mononuclear cells
PHQ-9	Patient health questionnaire 9
pNfL	Plasma neurofilament light chain
PSG	Polysomnography
RHI	Reactive hyperemia index
RT-qPCR	Reverse transcription quantitative polymerase chain reaction
SD	Standard deviation

## Appendix A

**Table A1:** Neuropsychological test battery for the assessment of OSA patients.

Test	Description	Time of Completion
Rey auditory—verbal learning test	A neuropsychological measure of verbal episodic memory, encompassing immediate recall, learning across trials, delayed recall, and recognition. Participants hear a list of 15 words across multiple trials and are asked to recall them immediately and after a delay [25].	15–20 min
Figure copy task	A visuoconstructional task from the Czech short neuropsychological battery. The subject is asked to precisely copy a geometric figure presented on a stimulus sheet. Administered twice (initial and retest) to assess visual perception, spatial planning, and memory [26].	5–10 min
Controlled oral word association test	A verbal fluency task requiring rapid generation of words beginning with specified letters (K—P—S for Slovak population). It assesses phonemic fluency, executive function, and lexical retrieval [27,28].	5 min
Category fluency test	A semantic fluency measure where subjects list as many words from a given category (e.g., animals) in one minute. It evaluates semantic memory and executive retrieval processes [27,28].	5 min
Trail making test	A two-part test evaluating processing speed, visual search, attention, and task-switching. Part A connects numbered dots; Part B alternates between numbers and letters [29].	5–7 min
Stroop color and word test	The stroop color and word test is a widely used neuropsychological measure of selective attention, processing speed, cognitive flexibility, and inhibitory control. In its classic form, participants complete three tasks: word reading, color naming and naming the ink color of color-words printed in an incongruent color (e.g., the word “RED” printed in blue ink), requiring the participant to inhibit the automatic tendency to read the word [30].	5 min
Digit span	A working memory test comprising forward and backward recall of digit sequences. Assesses attention, concentration, and short-term memory [31].	5 min
Montreal cognitive assessment (MoCA)	A 30-point screen for mild cognitive impairment targeting attention, executive function, memory, language, visuospatial abilities, and orientation [32].	10 min
Generalized anxiety disorder 7-item scale (GAD—7)	A 7-item self-report questionnaire to screen and assess the severity of generalized anxiety disorder. Items are rated on a 4-point scale [33].	3 min
Patient’s health questionnaire (PHQ—9)	A 9-item scale assessing depression severity based on DSM-IV criteria. Each item is rated from “not at all” to “nearly every day” [34].	2 min
Total assessment time		60–70 min
